# Effective pulmonary delivery of an aerosolized plasmid DNA vaccine via surface acoustic wave nebulization

**DOI:** 10.1186/1465-9921-15-60

**Published:** 2014-05-20

**Authors:** Anushi E Rajapaksa, Jenny J Ho, Aisha Qi, Rob Bischof, Tri-Hung Nguyen, Michelle Tate, David Piedrafita, Michelle P McIntosh, Leslie Y Yeo, Els Meeusen, Ross L Coppel, James R Friend

**Affiliations:** 1 Monash University, Department of Mechanical and Aerospace Engineering, Wellington Road, 3800 Clayton, Australia; 2 Monash University, Department of Chemical Engineering, Wellington Road, 3800 Clayton, Australia; 3 RMIT University, Micro Nano Research Facility, 124 La Trobe Street, 3000 Melbourne, Australia; 4 Melbourne Centre for Nanofabrication, 151 Wellington Road, 3800 Clayton, Australia; 5 Monash University, Monash Institute of Pharmaceutical Sciences, 3800 Parkville, Australia; 6 Monash University, Centre for Innate Immunity & Infectious Disease, Monash Institute of Medical Research, 3800 Clayton, Australia; 7 Monash University, Department of Physiology, School of Biomedical Sciences, Wellington Road, 3800 Clayton, Australia; 8 Monash University, School of Applied Sciences & Engineering, Wellington Road, 3800 Clayton, Australia; 9 Department of Physiology, School of Biomedical Sciences, Wellington Road, 3800 Clayton, Australia; 10 Monash University, Faculty of Medicine, Nursing and Health Sciences, Wellington Road, 3800 Clayton, Australia; 11 Critical Care and Neuroscience, Murdoch Children’s Research Institute, 3052 Parkville, Australia

**Keywords:** Gene therapy, Surface acoustic wave, Nebulization

## Abstract

**Background:**

Pulmonary-delivered gene therapy promises to mitigate vaccine safety issues and reduce the need for needles and skilled personnel to use them. While plasmid DNA (pDNA) offers a rapid route to vaccine production without side effects or reliance on cold chain storage, its delivery to the lung has proved challenging. Conventional methods, including jet and ultrasonic nebulizers, fail to deliver large biomolecules like pDNA intact due to the shear and cavitational stresses present during nebulization.

**Methods:**

*In vitro* structural analysis followed by *in vivo* protein expression studies served in assessing the integrity of the pDNA subjected to surface acoustic wave (SAW) nebulisation. *In vivo* immunization trials were then carried out in rats using SAW nebulized pDNA (influenza A, human hemagglutinin H1N1) condensate delivered via intratracheal instillation. Finally, *in vivo* pulmonary vaccinations using pDNA for influenza was nebulized and delivered via a respirator to sheep.

**Results:**

The SAW nebulizer was effective at generating pDNA aerosols with sizes optimal for deep lung delivery. Successful gene expression was observed in mouse lung epithelial cells, when SAW-nebulized pDNA was delivered to male Swiss mice via intratracheal instillation. Effective systemic and mucosal antibody responses were found in rats via post-nebulized, condensed fluid instillation. Significantly, we demonstrated the suitability of the SAW nebulizer to administer unprotected pDNA encoding an influenza A virus surface glycoprotein to respirated sheep via aerosolized inhalation.

**Conclusion:**

Given the difficulty of inducing functional antibody responses for DNA vaccination in large animals, we report here the first instance of successful aerosolized inhalation delivery of a pDNA vaccine in a large animal model relevant to human lung development, structure, physiology, and disease, using a novel, low-power (<1 W) surface acoustic wave (SAW) hand-held nebulizer to produce droplets of pDNA with a size range suitable for delivery to the lower respiratory airways.

## Background

The lung is an attractive site for delivery of gene therapy and DNA vaccine agents since it is accessible, has a large surface area and is highly cellular and vascularized to facilitate transfection. Further, pulmonary delivery via inhalation is non-invasive and allows for pain-free access where potential systemic side effects are minimized [1,2]. Replacement of the parenteral route with alternative modes of administration would mitigate vaccine safety issues and the requirement for skilled personnel, amongst the many other issues associated with injections [3,4]. Indeed, there has been significant interest to date in the potential of pulmonary gene therapy in treating pulmonary diseases caused by single gene mutation such as cystic fibrosis and 1-antitrypsin deficiency [5]. Further, there is potential for pulmonary vaccination to be useful against a range of pathogens, since immunity can be induced at mucosal sites through which these agents enter the body [2].

Effective pulmonary administration of DNA demonstrating cellular or tissue expression and subsequent induction of protective immunity has been a challenging task. Aerosols containing plasmid DNA (pDNA) can reach and adhere to the bronchial and alveolar epithelial cells only if the aerosol droplets are between 1 and 5 *μ*m, enabling pDNA entry and subsequent gene expression [6]. Recent work nevertheless suggests delivery via the mucosa may be more feasible than once thought [7].

While nebulizers are the delivery method of choice for macromolecules [6], delivery of non-complexed pDNA is impractical in current nebulizers due to poor droplet size control as well as the generation of sufficient hydrodynamic stresses that can shear pDNA molecules (>5 kbp) into open circular and fragmented configurations [8-10]. The transfection efficiency of such post-nebulized DNA has been shown to be as low as 10%, which often necessitates complexation of the DNA in an attempt to protect it from shear-induced degradation [11]. For example, the pulmonary delivery of cationic polymers has led to modestly improved gene expression in the airways of sheep [12,13]. However, not all polyplexes (nor lipoplexes) retain biological efficacy after aerosolization [14], with some commonly used synthetic polymers such as polyethylenimine (PEI) considered to be cytotoxic [15]. Effective delivery via the pulmonary route therefore requires the aerosolized DNA to be internalized into the target cell via endocytosis, avoiding degradation either during delivery or via exposure to lysosomal or cytoplasmic nucleases, and subsequent transcription and translation to produce the desired gene product [16].

Surface acoustic wave (SAW) nebulization was recently shown to effectively form aerosols of a short acting *β*_2_-agonist with a respirable fraction of >70% [17], much more than the typical 30–40% lung dose available via current nebulizers [18]. Rayleigh SAWs, transverse-axial polarized electroacoustic waves generated by a sinusoidal electric field between the interlaced fingers of an interdigital transducer (IDT) electrode, are formed and propagate at nanometer amplitudes at MHz to GHz-order frequencies along piezoelectric lithium niobate (LiNbO _3_). In such devices, the SAW is localized to the substrate surface, and most of the energy input into the system is near the surface and transferred into fluid resting upon it with minimal loss. As such, SAW nebulization requires only about 1 W of power to operate, significantly less than conventional bulk piezoelectric ultrasonic radiators, and convenient for use in handheld devices [19].

The aim of this study was to demonstrate the feasibility of SAW nebulization as an aerosol delivery platform for DNA delivery to the lungs in a large animal model.

## Methods

### Preparation of pDNA

The mouse malaria *P. yoelli* merozoite surface protein 4/5 (PyMSP4/5) was cloned into the mammalian expression vector pVR1020 and was used throughout the *in vitro* work [20]. The VR1020 plasmid encoding yellow florescent protein (YFP) that replaced the PyMSP4/5 gene was used for the *in vivo* studies to aid visualization of the gene expression. For the immunization trial, a plasmid DNA was prepared from a gene encoding an influenza A virus surface hemagglutinin protein, human hemagglutinin (A/Solomon Islands/3/2006 (egg passage) (H1N1) strain), once cloned into the mammalian expression vector pVR1020 (Vical Inc., USA). The entire coding sequence of HA was amplified by PCR using primers forward and reverse that incorporated a BamHI site at the 5’ end and a EcoRI site at the 3’ end, forward: 5’-CGCGGATCCATGAAAGTAAAACTACTGGTCCTGTTATG-3’; reverse: 5’-CCGGAATTCTTGTTTGTAATCCCATTAATGGCATTTTGT-3’. The PCR product was digested with BamHI/EcoRI and ligated into the vector, pVR1020, resulting the in plasmid pVR1020–HA.

A colony of *E. coli* DH5 *α* harboring either plasmid pVR1020–PyMSP4/5 (∼5.6 kbp), pVR1020–YFP (∼5.7 kbp) or pVR1020–HA (∼6 kbp) was picked from a streaked selective plate and inoculated in 10 ml of LB medium containing 100.0 *μ*g/ml of kanamycin. The starter culture was incubated at 37°C and agitated at 200 rpm for 8 h before being transferred to five separated 200 ml LB media, and further cultured for 12 h. The cell cultures were stored at -70°C for subsequent use. The plasmids were purified from cells using an endotoxin-free plasmid purification kit (QIAGEN Mega, Australia) according to the manufacturer’s instructions.

### SAW nebulization of Plasmid DNA

**Figure 1:**
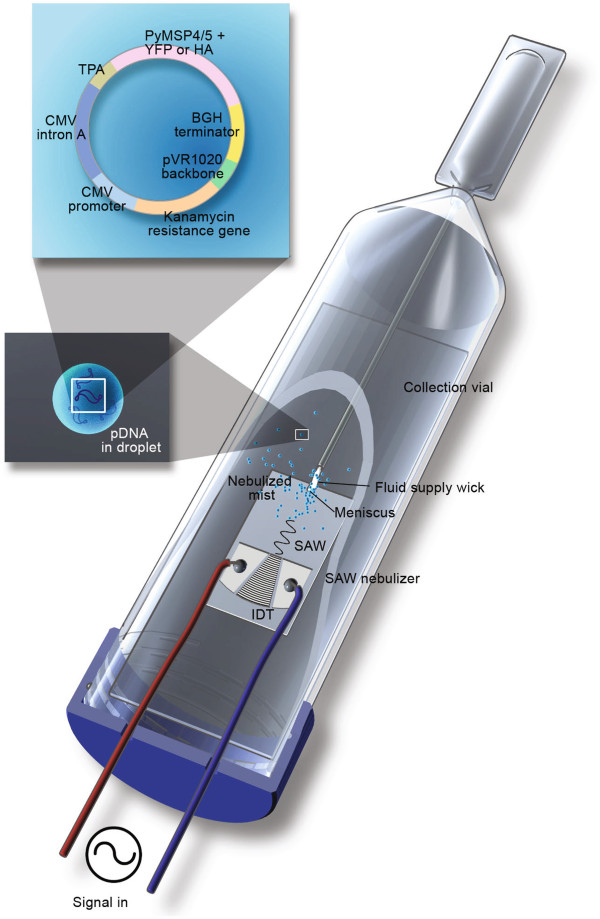
**Collecting nebulized pDNA from the SAW nebuliser.** A pDNA-laden meniscus forms at the end of the cellulose fiber wick upon the activation of the SAW nebulizer at the tip of the fiber wick and nebulizes inside the vial.

Different concentrations of pDNA solution (5–85 *μ*g/ml in deionized water for pVR1020–PyMSP4/5 and 1.5 mg/ml in 0.9% NaCl for pVR1020–YFP) were nebulized using both 20 and 30 MHz SAW devices at approximately 110 *μℓ*/min and carefully collected in microcentrifuge tubes for further analysis as illustrated in Figure 1. The concentration of pDNA in the collected samples was determined by a UV spectrophotometer (NanoDrop 1000, Thermo Scientific, USA) at a wavelength of 260 nm. The purity of pDNA samples was assessed by the samples’ absorbance at 260 and 280 nm.

### Agarose gel electrophoresis

Both control and nebulized pDNA samples were analyzed for potential alterations in the plasmid structure with 0.8% agarose gel electrophoresis that used a 1 kbp DNA ladder and ethidium bromide (EtBr) staining [21]. The gel was made up of 0.8 g agarose at 50 × dilution of TAE buffer (242.0 g Tris base, 57.1 ml CH3COOH, 9.3 g of EDTA). The gel was stained with 3 *μ*g/ml EtBr and the electrophoresis was carried out under 60 V for 90 min. The resulting gel was analyzed and imaged in a Molecular Imager Gel Doc XR system (Bio-Rad, Australia). The intensity of the bands for each structure corresponds to the number of DNA molecules. The percentage of supercoiled (SC) and open circular (OC) to degraded linear pDNA was quantified via densitometry software (Quantity One, Bio-Rad, Australia) by comparing pre- and post-nebulized samples. However, it was found that the binding of EtBr to different plasmid structures is dependent on the DNA topology, and a correction factor of 1.36 was therefore multiplied against the measured fluorescence values of the supercoiled structure [22].

### AFM imaging of pDNA

A freshly cleaved 10 mm diameter mica disc was coated with 10 *μ*l of 10 mM Ni(NO _3_) _2_ to render the mica surface cationic for adsorption of anionic pDNA. Ten *μ*l of pDNA solution (6–25 *μ*g/ml) was pipetted onto the mica surface. Two to five minutes were allowed to elapse to enable the pDNA to absorb onto the surface, after which the mica disc was rinsed with ultra-pure water and dried under a gentle stream of N _2_ gas prior to AFM imaging. The surface morphology of pDNA was imaged with an Ntegra Scanning Probe Laboratory (NT–MDT, Zelenograd, Russia) operating in intermittent contact (or tapping) mode in air using MikroMasch NSC15 probes [23]. Images of 512 × 512 pixels with a scan size of 3 × 3, 1 × 1 and 0.5 × 0.5 *μ*m^2^ were acquired at scan rates of 1–2 Hz. AFM images were processed to correct plane tilt, arcing and line fluctuations. For the presented images, 3 × 3 Gaussian noise filtering and 3D rendering was also applied using WsXM freeware (version 5, build 1.1, Nanotec Electronica S.L., Spain).

### Aerosol characterization

De Brouckere (volume moment, *D*_43_) mean diameter is the average particle size relevant to the dose’s delivery efficiency when the particles are droplets of unit density. To examine the aerosol size distribution of the nebulized pDNA, pVR1020–PyMSP4/5 was prepared in glycerol and the aerosol size for different preparations was characterized by laser diffraction (Spraytec, Malvern Instruments, UK) following nebulization using the SAW device. In order to characterize the pDNA aerosol size distribution, *D*_43_ was recorded during the measurements.

### Animal trials

For gene expression detection studies, male Swiss mice (8–10 week old) were used, weighing 37–40 g, and for the genetic immunization trial via intratracheal instillation, female Sprague-Dawley rats (8–10 week old), weighing 241–270 g, were purchased from Animal Resources Centre (Canning Vale, Australia). Mice and rats were housed under specific pathogen-free conditions at the Monash Institute of Pharmaceutical Sciences animal facility (Parkville, Australia) and had access to food and water *ad libitum*.

For the DNA vaccination of sheep via inhalation, female Merino-cross ewe lambs (5-6 months of age) used in these studies were housed in pens (Department of Physiology animal facility, Clayton, Australia) and fed *ad libitum* and judged free of significant pulmonary disease on the basis of clinical examination. All experimental animal procedures were approved by the Animal Experimentation Ethics Committee of Monash University, following guidelines set by the National Health and Medical Research Council (NHMRC) of Australia.

### Gene expression *in vivo* following intratracheal plasmid DNA delivery in mice

For *in vivo* transfection, the mice were anesthetized by intraperitoneal injection of 100 mg/kg body weight of ketamine (Parnell Laboratories, Australia) and 10 mg/kg body weight of xylazine (Tony Laboratories, Australia). A solution of sterile pVR1020 encoding YFP in 0.9% NaCl at a concentration of 1.5 mg/ml was nebulized using a 30 MHz SAW nebulizer and the condensed aerosol containing the nebulized plasmid was carefully collected as described earlier. For intratracheal instillation, the mice were suspended at 45 degrees by the upper teeth on a rodent dosing board and the trachea was visualized using a fiber optic stylet connected to an endotracheal tube (Biolite small animal intubation system, Kent Scientific Corp, USA). The trachea was intubated and post-nebulized plasmid in saline (50 *μ*l) was delivered followed by 200 *μ*l of air. The mice were sacrificed 24 hours later, and their lungs were harvested and subsequently frozen in Jung tissue freezing medium (Leica Microsystems, Germany) for later analysis of tissue sections. Cryosections (10 *μ*m) cut onto glass microscope slides were fixed with 1% paraformaldehyde, mounted with mowiol (4–88 Reagent from Calbiochem, Australia) solution to which 4’,6-diamidino–2–phenylindole dilactate (DAPI, dilactate) was added, and subsequently examined under a confocal laser scanning microscope (A1, Nikon Instruments Inc., Japan) for YFP gene expression. Lung and airway morphology was examined on adjacent sections with hematoxylin and eosin. As additional confirmation for the detection of YFP in the mice lungs, the lung samples were homogenized using lysis buffer containing 50 mM Tris (pH 7.5), 100 mM NaCl and 1% Triton X–100. The homogenate was then centrifuged and the supernatant was used for Western blot detection [24] of the YFP protein where SDS-PAGE and immunoblotting analysis procedures were carried out as previously described, except that the membranes were probed with rabbit anti-YFP antisera.

### Pulmonary intratracheal vaccinations using pre-SAW nebulized plasmid DNA in rats

For pulmonary vaccinations, solution of sterile pVR1020 encoding HA in 5% dextrose at a concentration of 300 *μ*g/ml was nebulized at approximately 110 *μ*l/min using a 30 MHz device and the condensed aerosol containing the nebulized plasmid was carefully collected as described earlier. Rat immunizations were carried out using the intratracheal instillation technique also described earlier where the trachea of the rats were intubated and 300 *μ*g of post-nebulized plasmid in 5% dextrose (*n* = 8, post-nebulized group), 300 *μ*g of pre-nebulized plasmid in 5% dextrose (*n* = 8, pre-nebulized group), and 5% dextrose (*n* = 8, naïve group) in a total volume of 100 *μ*l was delivered followed by 200 *μ*l of air. Subsequent immunizations were carried out 2 weeks (secondary) and 3 weeks (tertiary) after the primary immunization. Serum was collected prior to the commencement of the study and 5 weeks after the first immunization. Blood was collected from the tail vein, then left to coagulate for the collection of sera and stored at -20°C until further analyzed.

### Pulmonary DNA inhalation vaccination using SAW nebulization in sheep

**Figure 2:**
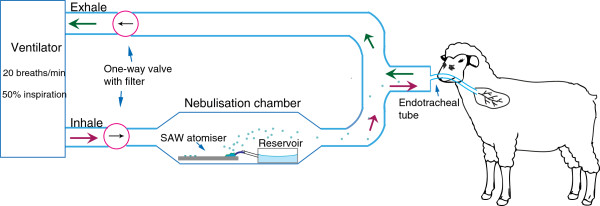
**Schematic drawing of the pulmonary delivery system used for aerosolized pDNA administration in conscious sheep via inhalation.** Room air was drawn through a bacterial/viral filter and a one-way valve placed in the inspiratory limb. Nebulized plasmid DNA was introduced into the inspiratory arm of the system via the 30 MHz SAW device positioned in a chamber in line with a mechanical ventilator to facilitate controlled respiration in sheep. The closed respiratory circuit was completed with the placement of an endotracheal tube via the nasal passage. Each sheep received three immunizations, once (20-30 minute aerosolization) per three-week period, with a pDNA aerosol containing sterile pVR1020 encoding HA (85 *μ*g/ml) in 5% dextrose.

Sheep (*n*=4) were immunized via inhalation through an endotracheal tube inserted through the nostril with sterile pVR1020 encoding HA in 5% dextrose at a concentration of 85 *μ*g/ml, nebulized using a 30 MHz SAW device in a chamber placed in line with the inspiratory limb of the mechanical ventilator (ventilator Model 55-0723; Harvard Apparatus, MA) set at 20 breaths/min at 50% inspiration for 20-30 minutes (as per Figure 2). Two bacterial/viral filters (Hudson RCI, USA) and two low flow resistance, Hudson one-way valves (Hudson RCI, USA) were placed at each end of the inspiratory and expiratory limbs in line with the rest of the connecting tubing. In order to calculate the delivered mass of pDNA to the ovine lung, all tubing, including the filters and valves, were carefully washed with deionized water to recoup any pDNA present, if any, and subsequently quantified via a UV spectrophotometer described earlier. Subsequent immunizations were carried out 3 weeks (secondary) and 6 weeks (tertiary) after the primary immunization. Serum from peripheral blood samples collected prior to the commencement of the study and 1 week after the last immunization were stored frozen prior to determination of hemagglutination inhibition activity.

### Evaluation of antibody responses by enzyme-linked immunosorbent assay

For the detection of anti-influenza (HA) antibodies in the serum samples, enzyme-linked immunosorbent assays (ELISAs) [25] were performed in 96-well MaxiSorp plates (Nunc, Denmark). Plates were coated with 0.05 *μ*g HA protein (Immune Technology Corp., USA) in carbonate coating buffer (0.015 M Na2CO _3_, 0.035 M NaHCO _3_, 0.003 M NaN _3_, pH 9.6) and blocked with 2% w/v skim milk powder. Serial 2-fold dilutions of serum samples were added in duplicate followed by anti-sheep IgG horseradish peroxidase (HRP) conjugated immunoglobulin (DAKO, Denmark) or dilution of anti-sheep IgA horseradish peroxidase (HRP) conjugated immunoglobulin (AbCam, Australia). Plates were developed with 3,3’,5,5’–tetramethylbenzidine (TMB) (Sigma Aldrich, Australia) substrate. Absorbance was measured at 450 nm and endpoint titers were calculated.

### Serum evaluation of hemagglutination inhibition activity

The serum samples collected from immunized sheep and rats were tested for inhibition activity against four hemagglutinating units (HAU) of A/Solomon Islands/3/2006 virus in microtiter plates at room temperature using 1% v/v chicken erythrocytes [26]. Virus-induced hemagglutination titers were determined as the minimum dilution of samples required to inhibit hemagglutinating activity of four HAU of virus.

### Statistical analysis

Statistical analyses were performed using SPSS (IBM Corporation, Armonk, USA). One-way ANOVA with a Tukey’s post-hoc test was used for data that survived Shapiro-Wilk’s (SW) normality test with significance *p*>0.05. In instances where the SW test was significant (*p*<0.05), the non-parametric Kruskal-Wallis (KW) test was used instead to test for the overall significance between independent groups. Where differences were observed, Mann-Whitney (MW) tests were performed between two independent samples to identify the differences. All data are expressed as the mean ± standard deviation. The results were considered significant if *p*<0.05.

## Results

### SAW nebulized pDNA displays aerosol size characteristics to suit deep lung deposition

**Figure 3:**
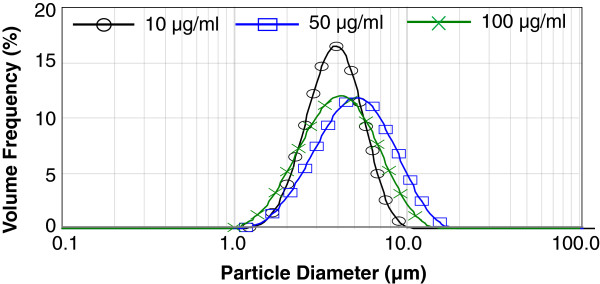
**Measured using laser diffraction (Spraytec, Malvern, UK), the size distribution of nebulized droplets using SAW at 30 MHz and 1 W of applied power from an aqueous suspensions of 10, 50 and** ***100 μ*** **g/ml of pDNA indicates droplet size distributions generally less than 5 *μ*** **m, useful for deep lung deposition.**

Our initial investigations sought to determine the aerosol size distribution of the nebulized pDNA (PyMSP4/5), prepared in glycerol, including the desired 1–5 *μ*m range for pulmonary delivery. As seen in Figure 3, the pDNA formulation at 100 *μ*g/ml with glycerol concentrations of 10%, 20% and 40% enabled average droplet size distributions under 5 *μ*m to be reliably achieved. Increasing the concentration of glycerol was seen to reduce the average aerosol diameter for a fixed pDNA concentration (Table 1), thus confirming our ability to establish some control over the desired aerosol dimension through the physical properties of the liquid.

**Table 1:** Mean aerosol droplet diameter for the SAW nebulizer

**pDNA concentration (*μ*** **g/ml)**	**% w/w glycerol**	***D***_***43 ***_**(*μ*** **m)**
100	0	8.25±0.34
	10	7.61±0.94
	20	4.12±0.13
50	20	5.49±0.10
10	20	4.08±0.02

### SAW nebulization preserves integrity of pDNA

**Figure 4:**
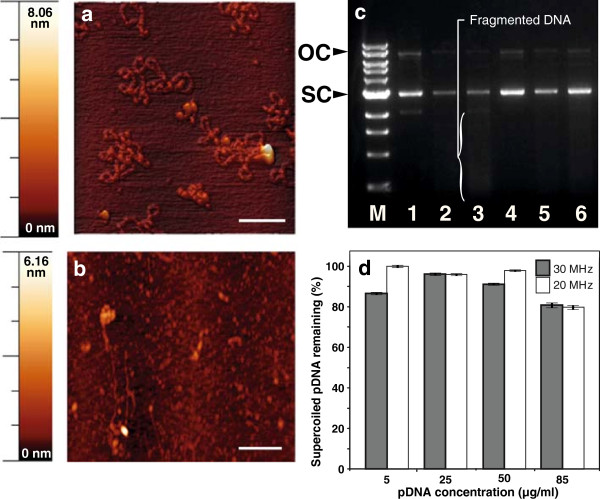
**SAW nebulization preserves the integrity of pDNA.** Atomic force microscopy **(a, b)** and ethidium bromide agarose gel electrophoresis **(c)** of pVR1020–PyMSP4/5 **(a)** before and **(b)** after nebulization. Lane M: 1 kbp DNA ladder; control lanes 1 and 4, recovered pDNA post-30 MHz lanes 2 and 5, and post-20 MHz SAW nebulization lanes 3 and 6 are each at 85 and 50 *μ*g/ml concentrations, respectively. The proportion of **(d)** supercoiled (SC), open circular (OC) and fragmented pDNA quantifies the damage (*n*=3). Bars represent the mean of trplicate samples with error bars indicating the standard error of the mean. Data is representative of three independent experiments.

The structural integrity of pDNA plays a key role in preserving the bio-activity where AFM imaging and agarose gel electrophoresis was used for structural analysis of pDNA encoding PyMSP4/5 before and after SAW nebulization. AFM imaging revealed that the non-nebulized plasmid DNA showed a tightly twisted supercoiled geometry, with few relaxed strands (open circular structures) (Figure 4(a)), characteristic morphologies for uncondensed plasmids [27]. A representative image of the SAW nebulized pDNA showed some aggregated structures and some relaxed open loop structures with little fragmented strands (Figure 4(b)). These structural characteristics were further confirmed upon examination of the pDNA preparations by agarose gel electrophoresis, where changes in the supercoiled, open circular and fragmented pDNA structures prior to and after nebulization were observed (Figure 4(c)). Significantly, the unprotected naked pDNA was preserved during the SAW nebulization process, and in most cases more than 90% of the initial supercoiled pDNA was still present post-nebulization (Figure 4(d)).

### Airway administration of nebulized pDNA induces gene expression in the murine lung

**Figure 5:**
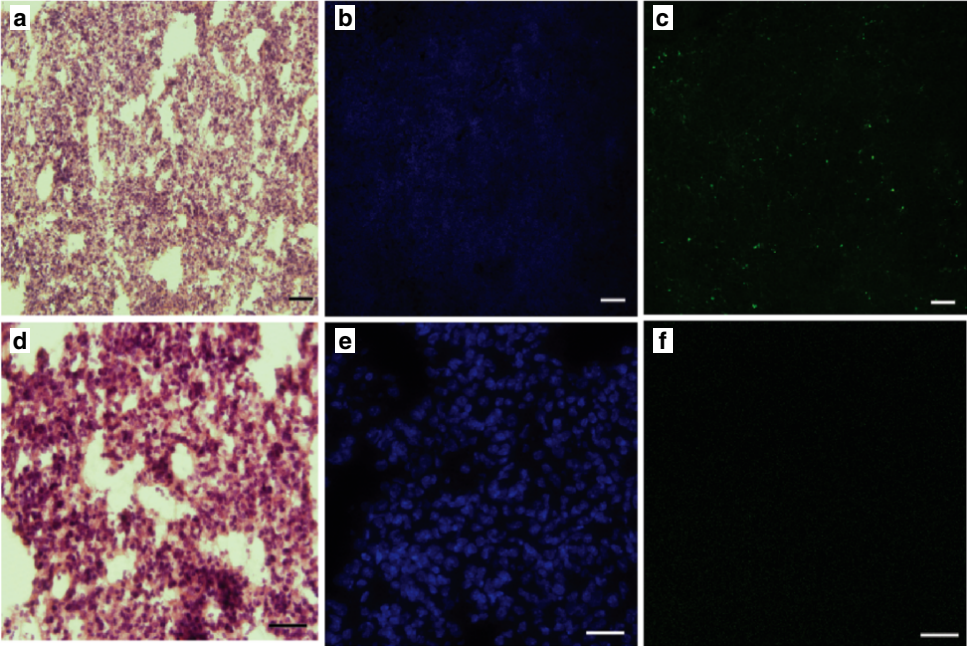
**Administration of nebulized pDNA induces gene expression in the murine lung.** Confocal microscopy of mouse lung parenchyma cryosections 24 hours after **(a, b, c)** dosage of 75 *μ*g with post-nebulized pVR1020–YFP in 0.9% NaCl aqueous solution, compared to an **(d, e, f)** untreated case (scale bars: 100 *μ*m). The lung structure **(a, d)** and **(b, e)** cell nuclei are indicated with a counterstain of hematoxylin and eosin, and with DAPI, respectively. Lung cells expressing YFP from instilled pVR1020–YFP appear green in **(c)** the treated lung sample; note the absence of green in the **(f)** untreated sample. The control lung samples were imaged at a higher resolution to confirm the absence of YFP response.

**Figure 6:**
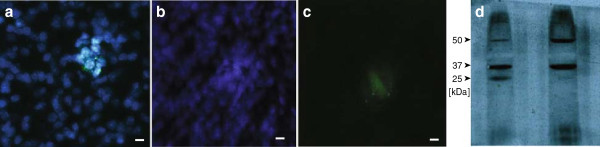
**YFP is expressed in the epithelial cells of the terminal airways.** YFP expression patterns observed using confocal microscopy of mouse lung parenchyma cryosections 24 hours after **(a, b and c)** dosage of 75 *μ*g with post-nebulized pVR1020–YFP in 0.9% NaCl aqueous solution showing **(a)** YFP expression in a cluster of epithelial cells where lung cell nuclei stained with DAPI dilactate appear blue while cells expressing YFP appear green (scale bar =5 *μ*m); **(b)** another view of a part of the YFP-expressing cell region shows the YFP distribution in the cells with appropriate filtering to **(b)** show only the nuclei and **(c)** YFP (scale bar =5 *μ*m). **(d)** Western blot of the supernatant obtained from homogenized mice lungs harvested (left lane) 24 hours post-transfection with 300 *μ*g pVR1020–YFP plasmid that was SAW nebulized at 30 MHz and instilled, compared to (right lane) the supernatant from untreated mice lungs. The YFP protein appears clearly at 27 kDa. The additional protein bands present at 50 and 37 kDa for both groups are due to the polyclonal nature of the rabbit anti-YFP antisera used as the probe where cross-reactivity was induced across other serum proteins.

Mice were used to assess gene expression following SAW-nebulized airway administration of pDNA encoding yellow fluorescent protein (YFP) (Figure 5(a)-(f)). Scattered, indiscriminate YFP expression was observed in the mouse lung when examined 24 hours following intratracheal instillation with condensed nebulized pVR1020-YFP (Figure 5(c)). Importantly, YFP expression was absent in the untreated mouse lung (Figure 5(f)). YFP-positive cells detected in the mouse lung were mainly located close to the epithelium of the conducting airways, where there appeared to be discrete aggregates of YFP protein (Figure 6(a)-(c)). Further confirmation of YFP expression in the murine lung was demonstrated in Western blot analysis of lung tissue collected 24 hours post-pDNA administration (Figure 6(d)).

No tissue damage was evident in the mouse lungs instilled with SAW-nebulized pDNA (as indicated by the absence of hemosiderin deposits in tissue sections stained with Perl ^′^s Prussian blue) and was confirmed by the lack of inflammatory cells and lack of micro-haemorrhaging in the lung tissue (data not shown).

### Lung pDNA vaccination induces serum antibody responses and HAI activity in rats

**Figure 7:**
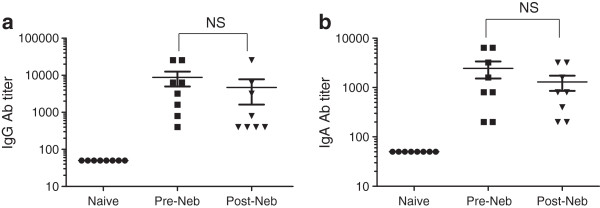
**Pulmonary delivery of pVR1020-HA pDNA induces antibody response in rats.** Systemic and mucosal **(a)** IgG and **(b)** IgA antibody responses detected in the sera of female Sprague-Dawley rats (*n*=8 per group) 3 weeks following 300 *μ*g pVR1020-HA pDNA vaccination (primary immunization) encoding an influenza A virus surface antigen, human hemagglutinin (HA) via lung instillation. Bars represent the mean ± SD and individual rats are indicated as symbols. No significant (NS) difference between the pre and post-SAW nebulized vaccine instillation was found (*p*=0.163 for IgG and *p*=0.486 for IgA, respectively), and a significant difference between these and the naïve rat was found (*p*<0.001). Note that the antibody response detected in the naïve rat group represents the detection limit of the assay.

Sprague Dawley rats were used as a suitable small animal model for infuenza [28]. To examine antibody responses to pDNA vaccination (pVR1020 encoding HA) via intratracheal instillation in rats, ELISAs were performed on sera collected prior to and two weeks after a third airway immunization. Significant increases in both IgG and IgA antibody titer levels against the influenza virus HA protein were observed in both groups of rats that received pre-nebulized and post-nebulized pDNA vaccinations, compared to the vehicle-only control group (Figure 7, *p*<0.001). Importantly, no differences in IgG or IgA levels (*p*>0.05 in both cases) were observed between the groups that received the nebulized and non-nebulized form of the pDNA vaccine.

Antibody responses detected in sera following lung instillation with the pDNA vaccine showed clear functional activity in the hemagglutination inhibition (HAI) assay (Table 2). Both groups that received pDNA vaccine achieved appropriate HAI titers at levels considered to be protective by the World Health Organization (WHO) [29] in establishing immunity in humans. Compared to the control group, serum from rats immunized with pDNA via intratracheal instillation showed significantly increased HAI activity (*p*<0.001), with no difference seen between the pre-nebulized and post-nebulized forms of pDNA.

**Table 2:** Hemagglutination inhibition activity after lung immunization of rats

**Rat**	**Naïve**		**Pre-nebulized**		**Post-nebulized**	
**No.**	**Pre**	**Post**	**Pre**	**Post**	**Pre**	**Post**
	**Vaccination**	**Vaccination**	**Vaccination**	**Vaccination**	**Vaccination**	**Vaccination**
	**Titer**	**Titer**	**Titer**	**Titer**	**Titer**	**Titer**
1	<5	<5	<5	800	<5	800
2	<5	<5	<5	1600	<5	800
3	<5	<5	<5	1600	<5	800
4	<5	<5	<5	1600	<5	1600
5	<5	<5	<5	1600	<5	1600
6	<5	<5	<5	1600	<5	1600
7	<5	<5	<5	1600	<5	1600
8	<5	<5	<5	1600	<5	1600

### Aerosolized pDNA vaccination induces antibodies in sheep

Sheep were used to assess the efficacy of SAW-nebulized airway administration of pDNA in a large animal model with similar lung physiology to humans [30]. The sheep were administered three airway immunizations at three-week intervals using a 30 MHz SAW device, nebulizing the pDNA solution at approximately 110 *μℓ*/min. The average dose of pDNA delivered to the airways was 156 ±44 *μ*g (mean ± S.D.; data not shown). SAW-nebulized pDNA delivered to the sheep airways induced significant HAI activity, with a mean HAI titer of 192 ±74 (*n*=4), in sera collected one week after the third immunization (Table 3), and was comparable to the outcome observed in rats (see Table 2).

**Table 3:** Hemagglutination inhibition activity after pulmonary aerosol immunization of sheep

**Sheep**	**Pre**	**Post**
**No.**	**Vaccination**	**Vaccination**
	**Titer**	**Titer**
1	<1	256
2	<1	128
3	<1	256
4	<1	128

## Discussion

In this study, we demonstrate the use of a SAW nebulization device for the generation of aerosolized pDNA with suitable size and stability characteristics to facilitate effective pulmonary delivery particularly for influenza vaccination. The *in vivo* studies conducted showed for the first time, successful delivery of the SAW-generated pDNA to the airways of mice, rats, and importantly, in a large animal model (sheep).

### Generation of SAW nebulized droplets effective for deep lung deposition

The generation of aqueous pDNA aerosol droplets with sizes smaller than the 5 *μ*m diameter required for optimal deposition in the deep lung region [31] is particularly difficult due to the high surface tension of water [19]. The nebulized droplet size is independent of excitation frequency, but strongly dependent on fluid characteristics to provide a route to effectively tune droplet size [17]. During SAW nebulization, the droplet size formed from the device is governed by the wavelength of the capillary waves generated on the surface of the source drop [19]. The wavelength in turn, is predicted by the balance between the capillary and viscous forces that dominate at the surface such that the droplet diameter could be lowered by increasing the surface tension and dynamic viscosity of the source drop [19]. In the present study, glycerol, known to be safe in aerosol form [32], was introduced to modify the viscosity. The concentration of the pDNA had a minor effect on the droplet diameter that can be addressed by changing the glycerol concentration. By employing 20% glycerol in the pDNA stock solution, the SAW nebulizer delivered droplets with a mean diameter less than 5 *μ*m, which is required for effective deep lung deposition.

### SAW nebulization preserves the DNA integrity

Compact supercoiled DNA is the most immunogenic form and, according to FDA requirements, must be present at >80% in a licensable vaccine [33,34]. The SAW nebulization process is verified in this study to preserve the DNA integrity, where more than 90% of the initial supercoiled pDNA was still present after SAW nebulization. Scission of one of the two strands of the pDNA releases torsional energy stored in the supercoiled plasmid, and causing it to relax into an open circular form, while scission of both strands resuls in a linear polynucleotide and subsequent DNA fragments [35]. AFM and ethidium bromide agarose gel electrophoresis of the pDNA (pVR1020–PyMSP4/5) before and after SAW nebulization showed that the tightly twisted supercoiled geometry of the pDNA was effectively maintained. The AFM images nevertheless showed some morphological differences between the non-nebulized and SAW-nebulized pDNA, where the later showed aggregated structures commonly induced during nebulization processes [36], presumed to be tightly twisted strands of pDNA. The frequency of the SAW nebulizer had little effect on the results, and likewise, pDNA concentrations in the source drop prior to nebulization (5 to 85 *μ*g/ml) had minimal effect on the percentage of supercoiled pDNA left after nebulization.

A small proportion of the pDNA prior to SAW nebulization was found to be fragmented, most likely occurring during plasmid purification and preparation. This was especially evident at a pDNA concentration of 5 *μ*g/ml, with around 50% and 20% of supercoiled and fragmented pDNA, respectively. Linear, double-stranded DNA is very susceptible to flow-induced stresses. For example, minor differences in their characteristic dimension that arise from scission have large effects on their response to hydrodynamic stresses [37]. Stretching hydrodynamic forces of only 0.3 nN, well below the 1.6–5.0 nN required to break the covalent bonds within, are known to cause irreversible strand separation and formation of nicks, degrading the pDNA [9]. Though cavitation is absent in SAW nebulization [19], accelerations in the fluid of 10 ^7^–10 ^8^ m/s ^2^ are typically due to the MHz-order frequencies used. If the relaxed forms of the pDNA are adjacent to the fluid interface, it is possible that the gradient in the acceleration and consequently the shear stress across the pDNA molecules is sufficient to cause further damage, though the relaxation time scale of the fluid shear, ∼10 ns, is two orders of magnitude smaller than that required to shear large molecules, suggesting the risk of denaturing molecules such as DNA [38] is negligible. Compared to standard ultrasonic nebulization at 20–80 kHz, where cavitation is prevalent and large molecules like pDNA in solution actually serve as cavitation bubble nucleation sites [10] and cause wholesale destruction of such molecules, the damage caused by SAW nebulization is minor. For 5 kbp plasmids, the percentage of fragmented pDNA after nebulization is lower with SAW (<20%) than with conventional (>35%) or vibrating mesh nebulizers (>40%) [39]. Hence, the SAW nebulization approach has several key advantages over the current generation of ultrasonic medical nebulizers for the delivery of large molecules [19].

We set out to test the capacity of nebulized DNA to induce immune responses *in-vivo*. Previous studies using small animal models to investigate aerosol delivery of pDNA formulations have found it difficult to effectively control the delivered dose via nebulization [40]. Hence preliminary experiments in these animals involved recovery of the nebulized material followed by intratracheal instillation of the condensate. When post-nebulized pVR1020–YFP plasmid was introduced into the lungs of mice, the expression of yellow fluorescent protein (YFP) provided evidence of transfection that was entirely absent in the lungs of untreated mice. We hypothesize that the expression of YFP which appears as discrete aggregates with granular appearance was presumably close to the epithelium of the conducting airways, consistent with gene expression patterns following DNA vaccination in mouse lungs in another study [12]. Furthermore, Western blot analysis confirmed the presence of the YFP only in the lungs of treated lungs.

### SAW nebulized DNA induces protective antibodies after pulmonary delivery

More importantly, our data showed that SAW nebulization did not inhibit the ability of the vaccine to induce protective antibodies. Anti-HA antibody titers detected here were found to be comparable to the vaccination outcomes using a similar pDNA influenza vaccine complexed with polyethyleimine (PEI) [12]. Our results suggest that naked pDNA can be effectively delivered to the lung with subsequent transfection into airway cells (despite likely degradation of the pDNA) while also demonstrating preserved immunogenicity of the DNA vaccine subjected to the SAW nebulization process.

Following pDNA vaccination in rats, serum hemagglutination inhibition (HAI) titers were significantly greater than 40, levels considered to be protective according to WHO standards [29] and indicative of what would translate into a significant humoral response elicited to administered pDNA. These results are particularly encouraging when compared elsewhere to the outcome of vaccination with pDNA encoding swine H1N1 complexed with PEI and administered intranasally to BALB/c mice [41]. Although low doses of pDNA were used in this study, some synthetic adjuvants such as PEI are known to be highly cytotoxic [15], and are likely to encounter problems with approval for human use. Our results in sheep also compare well to the administration of HA protein in sheep via subcutaneous injection and via intratracheal instillation to the lung that resulted in HAI titers of 95 to 122 [42].

Translation of successful outcomes with DNA vaccination seen in small (rodent) to larger animal models [43] including humans [44] continues to represent a major hurdle in this emerging field. The similarities in size, structure and physiology of sheep and human lungs [30] suggests that sheep could be used as a relevant large animal model to test the efficacy of pulmonary DNA vaccination for human applications. In the present study, the SAW nebulized pDNA induced a robust antibody response when delivered into the lungs of conscious sheep under mechanical respiration, with similar levels in antibody response to that observed in rats. Given the scope of the current study, we have not included the impactor measurement data. However, the sheep aerosol study confirms that the aerosols generated by SAW were indeed suited for deep lung delivery (droplet size range 0.5-5 *μ*m), evidenced by the HAI assays and the induction of the functional antibodies.

This study reports the first instance of successful pDNA delivery via inhalation using an unprotected pDNA vaccine in a clinically relevant large animal model. Due to similarities with humans in lung structure and physiology, sheep provide a better preclinical model than smaller animals for the optimization of nebulizers intended for lung gene delivery. Studies presented here and elsewhere indicate that sheep are proving to be an invaluable model for the assessment of gene therapy efficacy, the delivery profile of the nebulizer to suit conventional sized devices and the safety of the gene transfer protocol, all critically relevant endpoint measurements [45]. Further, the present findings suggest that the SAW nebulizer may serve to effectively deliver pDNA vaccines that can be rapidly produced upon demand. This represents a significant outcome in the context of ensuring a timely response to pandemic disease outbreaks, and in the developing world where there is neither a sufficient health workforce nor access to safe injection methods.

## Competing interests

The authors declare that they have no competing interests.

## Authors’ contributions

JF and LY designed and developed the nebulizer technology, designed the experiments with EM and RC, and supervised the work. AR, JH, and AQ conducted the experiments, compiled the results, conducted the statistical analysis, and wrote the initial drafts of the manuscript. AR, AQ, RB, LY, RC and JF wrote the final draft of the manuscript. MP, THN, DP and EM aided with the rat and mouse model experiments. DP, RB and EM also aided with the sheep model experiments, and RC provided pDNA samples and provided input to AR, JH and AQ on their analysis of the pDNA. All authors read and approved the final manuscript.

## References

[B1] Forde GM **Rapid-response vaccines-does DNA offer a solution?** Nat Biotechnol 2005 23 9 1059 1062 10.1038/nbt0905-1059 16151391 PMC7097425

[B2] Birchall J **Pulmonary delivery of nucleic acids** Expert Opin Drug Deliv 2007 4 6 575 578 10.1517/17425247.4.6.575 17970661

[B3] Giudice EL Campbell JD **Needle-free vaccine delivery** Adv Drug Deliv Rev 2006 58 1 68 89 10.1016/j.addr.2005.12.003 16564111

[B4] Simonsen L Kane A Lloyd J Zaffran M Kane M **Unsafe injections in the developing world and transmission of bloodborne pathogens: a review** Bull World Health Organ 1999 77 10 789 800 10593026 PMC2557743

[B5] West J Rodman DM **Gene therapy for pulmonary diseases** Chest 2001 119 2 613 617 10.1378/chest.119.2.613 11171744

[B6] Lu D Hickey AJ **Pulmonary vaccine delivery** Expert Rev Vaccines 2007 6 2 213 226 10.1586/14760584.6.2.213 17408371

[B7] Ye L Zeng R Bai Y Roopenian D. C Zhu X **Efficient mucosal vaccination mediated by the neonatal Fc receptor** Nat Biotechnol 2011 29 2 158 163 10.1038/nbt.1742 21240266 PMC3197702

[B8] Catanese D Fogg J Schrock D Gilbert B Zechiedrich L **Supercoiled minivector DNA resists shear forces associated with gene therapy delivery resists shear forces associated with gene therapy delivery** Gene Ther 2011 19 1 94 100 21633394 10.1038/gt.2011.77 PMC3252587

[B9] Arulmuthu ER Williams DJ Baldascini H Versteeg HK Hoare M **Studies on aerosol delivery of plasmid DNA using a mesh nebulizer** Biotechnol Bioeng 2007 98 5 939 955 10.1002/bit.21493 17497741

[B10] Lentz Y Anchordoquy T Lengsfeld C **DNA acts as a nucleation site for transient cavitation in the ultrasonic nebulizer** J Pharm Sci 2006 95 3 607 619 10.1002/jps.20511 16432878

[B11] Birchall JC Kellaway IW Gumbleton M **Physical stability and in-vitro gene expression efficiency of nebulised lipid-peptide-DNA complexes** Int J Pharm 2000 197 1-2 221 231 10.1016/S0378-5173(00)00339-2 10704809

[B12] Davies LA McLachlan G Sumner-Jones SG Ferguson D Baker A Tennant P Gordon C Vrettou C Baker E Zhu J Alton EWFW Collie DDS Porteous DJ Hyde SC Gill DR **Enhanced lung gene expression after aerosol delivery of concentrated pDNA/PEI complexes** Mol Ther 2008 16 7 1283 1290 10.1038/mt.2008.96 18500249

[B13] McLachlan G Baker A Tennant P Gordon C Vrettou C Renwick L Blundell R Cheng SH Scheule RK Davies L Painter H Coles RL Lawton AE Marriott C Gill DR Hyde SC Griesenbach U Alton EWFW Boyd AC Porteous DJ Collie DDS **Optimizing aerosol gene delivery and expression in the ovine lung** Mol Ther 2006 15 2 348 354 17235313 10.1038/sj.mt.6300058

[B14] Rudolph C Ortiz A Schillinger U Jauernig J Plank C Rosenecker J **Methodological optimization of polyethylenimine (PEI)-based gene delivery to the lungs of mice via aerosol application** J Gene Med 2005 7 1 59 66 10.1002/jgm.646 15538727

[B15] Moghimi SM Symonds P Murray JC Hunter AC Debska G Szewczyk A **A two-stage poly (ethylenimine)-mediated cytotoxicity: implications for gene transfer/therapy** Mol Ther 2005 11 6 990 995 10.1016/j.ymthe.2005.02.010 15922971

[B16] Banks G Roselli R Chen R Giorgio T **A model for the analysis of nonviral gene therapy** Gene Ther 2003 10 20 1766 1775 10.1038/sj.gt.3302076 12939643

[B17] Qi A Friend JR Yeo LY Morton DAV McIntosh MP Spiccia L **Miniature inhalation therapy platform using surface acoustic wave microfluidic atomization** Lab Chip 2009 9 15 2184 2193 10.1039/b903575c 19606295

[B18] Clay MM Clarke SW **Wastage of drug from nebulisers: a review** J R Soc Med 1987 80 1 38 39 3550079 10.1177/014107688708000115 PMC1290632

[B19] Qi A Yeo LY Friend JR **Interfacial destabilization and atomization driven by surface acoustic waves** Phys Fluids 2008 20 074103 10.1063/1.2953537

[B20] Wang L Kedzierski L Schofield L Coppel RL **Influence of glycosylphosphatidylinositol anchorage on the efficacy of DNA vaccines encoding plasmodium yoelii merozoite surface protein 4/5** Vaccine 2005 23 32 4120 4127 10.1016/j.vaccine.2005.03.016 15964480

[B21] Lentz Y Worden L Anchordoquy T Lengsfeld C **Effect of jet nebulization on DNA: Identifying the dominant degradation mechanism and mitigation methods** J Aerosol Sci 2005 36 8 973 990 10.1016/j.jaerosci.2004.11.017

[B22] Projan SJ Carleton S Novick RP **Determination of plasmid copy number by fluorescence densitometry** Plasmid 1983 9 2 182 190 10.1016/0147-619X(83)90019-7 6344110

[B23] Glover DJ Ng SM Mechler A Martin LL Jans DA **Multifunctional protein nanocarriers for targeted nuclear gene delivery in nondividing cells** FASEB J 2009 23 9 2996 3006 10.1096/fj.09-131425 19395475

[B24] Korfhagen TR Bruno MD Ross GF Huelsman KM Ikegami M Jobe AH Wert SE Stripp BR Morris RE Glasser SW Bachurski CJ Iwamoto HS Whitsett JA **Altered surfactant function and structure in sp-a gene targeted mice** Proc Natl Acad Sci 1996 93 18 9594 9599 10.1073/pnas.93.18.9594 8790375 PMC38473

[B25] Haan Ld **Nasal or intramuscular immunization of mice with influenza subunit antigen and the B subunit of Escherichia coli heat-labile toxin induces IgA- or IgG-mediated protective mucosal immunity** Vaccine 2001 19 20-22 2898 2907 10.1016/S0264-410X(00)00556-9 11282201

[B26] Vujanic A Snibson KJ Wee JLK Edwards SJ Pearse MJ Scheerlinck J-PY Sutton P **Long-term antibody and immune memory response induced by pulmonary delivery of the influenza iscomatrix vaccine** Clin Vaccine Immunol 2012 19 1 79 83 10.1128/CVI.05265-11 22072721 PMC3255945

[B27] Hansma HG **Surface biology of DNA by atomic force microscopy** Annu Rev Phys Chem 2001 52 71 92 10.1146/annurev.physchem.52.1.71 11326059

[B28] Daniels MJ Selgrade MK Doerfler D Gilmour MI **Kinetic profile of influenza virus infection in three rat strains** Comp Med 2003 53 3 293 298 12868575

[B29] Richie TL Saul A **Progress and challenges for malaria vaccines** Nature 2002 415 6872 694 701 10.1038/415694a 11832958

[B30] Meeusen EN Snibson KJ Hirst SJ Bischof RJ **Sheep as a model species for the study and treatment of human asthma and other respiratory diseases** Drug Discov Today: Dis Models 2009 6 4 101 106 10.1016/j.ddmod.2009.12.002

[B31] Barnes P **New treatments for chronic obstructive pulmonary disease** Curr Opin Pharmacol 2001 1 217 222 10.1016/S1471-4892(01)00039-X 11712742

[B32] Renne RA Wehner AP Greenspan BJ Deford HS Ragan HA Westerberg RB Buschbom RL Burger GT Hayes AW Suber RL **Mosberg AT: 2-week and 13-week inhalation studies of aerosolized glycerol in rats** Inhal Toxicol: Int Forum Respir Res 1992 4 2 95 111 10.3109/08958379209145307

[B33] Klinman DM Klaschik S Tross D Shirota H Steinhagen F **FDA guidance on prophylactic DNA vaccines: analysis and recommendations** Vaccine 2010 28 16 2801 2805 10.1016/j.vaccine.2009.11.025 19941989 PMC2847045

[B34] Cai Y Rodriguez S Hebel H **DNA vaccine manufacture: scale and quality** Expert Rev Vaccines 2009 8 9 1277 91 10.1586/erv.09.84 19722898

[B35] Lengsfeld C Anchordoquy T **Shear-induced degradation of plasmid DNA** J Pharm Sci 2002 91 7 1581 1589 10.1002/jps.10140 12115820

[B36] Kleemann E Dailey L Abdelhady H Gessler T Schmehl T Roberts C Davies M Seeger W Kissel T **Modified polyethylenimines as non-viral gene delivery systems for aerosol gene therapy: investigations of the complex structure and stability during air-jet and ultrasonic nebulization** J Control Release 2004 100 3 437 450 10.1016/j.jconrel.2004.09.005 15567508

[B37] Levy M Collins I Yim S Ward J Titchener-Hooker N Ayazi Shamlou P Dunnill P **Effect of shear on plasmid DNA in solution** Bioprocess Biosyst Eng 1999 20 1 7 13 10.1007/s004490050552

[B38] Hsieh CC Balducci A Doyle PS **An experimental study of DNA rotational relaxation time in nanoslits** Macromolecules 2007 40 14 5196 5205 10.1021/ma070570k

[B39] Lentz YK Anchordoquy TJ Lengsfeld CS **Rationale for the selection of an aerosol delivery system for gene delivery** J Aerosol Med 2006 19 3 372 384 10.1089/jam.2006.19.372 17034312

[B40] Köping-Höggård M **A miniaturized nebulization catheter for improved gene delivery to the mouse lung** J Gene Med 2005 7 9 1215 1222 10.1002/jgm.762 15895386

[B41] Torrieri-Dramard L Lambrecht B Ferreira H. L Van den Berg T Klatzmann D Bellier B **Intranasal DNA vaccination induces potent mucosal and systemic immune responses and cross-protective immunity against influenza viruses** Mol Ther 2010 19 3 602 611 20959813 10.1038/mt.2010.222 PMC3048174

[B42] Wee J Scheerlinck JPY Snibson K Edwards S Pearse M Quinn C Sutton P **Pulmonary delivery of ISCOMATRIX influenza vaccine induces both systemic and mucosal immunity with antigen dose sparing** Mucosal Immunol 2008 1 6 489 496 10.1038/mi.2008.59 19079216

[B43] Babihk LA Pontarollo R Babiuk S Loehr B **van Drunen Littel-van den Hurk S: Induction of immune responses by DNA vaccines in large animals** Vaccine 2003 21 1 649 658 12531334 10.1016/s0264-410x(02)00574-1

[B44] Shedlock DJ Weiner DB **DNA vaccination: antigen presentation and the induction of immunity** J Leukoc Biol 2000 68 6 793 806 11129646

[B45] McLachlan G Davidson H Holder E Davies LA Pringle IA Sumner-Jones SG Baker A Tennant P Gordon C Vrettou C Blundell R Hyndman L Stevenson B Wilson A Doherty A Shaw DJ Coles RL Painter H Cheng SH Scheule RK Davies JC Innes JA Hyde SC Griesenbach U Alton EW Boyd AC Porteous DJ Gill DR Collie DD **Pre-clinical evaluation of three non-viral gene transfer agents for cystic fibrosis after aerosol delivery to the ovine lung** Gene Ther 2011 18 10 996 1005 10.1038/gt.2011.55 21512505

